# Exploiting heterogeneous features to improve *in silico* prediction of peptide status – amyloidogenic or non-amyloidogenic

**DOI:** 10.1186/1471-2105-12-S13-S21

**Published:** 2011-11-30

**Authors:** Smitha Sunil Kumaran Nair, NV Subba Reddy, KS Hareesha

**Affiliations:** 1 Department of Computer Science and Engineering, Manipal Institute of Technology, Manipal University, Karnataka, India; 2 Mody Institute of Technology and Science University, Rajasthan, India

## Abstract

**Background:**

Prediction of short stretches in protein sequences capable of forming amyloid-like fibrils is important in understanding the underlying cause of amyloid illnesses thereby aiding in the discovery of sequence-targeted anti-aggregation pharmaceuticals. Due to the constraints of experimental molecular techniques in identifying such motif segments, it is highly desirable to develop computational methods to provide better and affordable *in silico* predictions.

**Results:**

Accurate *in silico* prediction techniques of amyloidogenic peptide regions rely on the cooperation between informative features and classifier design. In this research article, we propose one such efficient fibril prediction implementation exploiting heterogeneous features based on bio-physio-chemical (BPC) properties, auto-correlation function of carefully selected amino acid indices and atomic composition within a protein fragment of amino acids in a window. In an attempt to get an optimal number of BPC features, an evolutionary Support Vector Machine (SVM) integrating a novel implementation of hybrid Genetic Algorithm termed Memetic Algorithm and SVM is utilized. Five prediction modules designed using Artificial Neural Network (ANN) models are trained with independent and integrated features in order to validate the fibril forming motifs. The results provide evidence that incorporating new feature namely auto-correlation function besides BPC, attempt to strengthen the sequence interaction effect in forming the feature vector thereby obtaining better prediction quality in terms of sensitivity, specificity, Mathews Correlation Coefficient and Area under the Receiver Operating Characteristics curve.

**Conclusion:**

A significant improvement in performance is observed by introducing features like auto-correlation function that maintains sequence order effect, in addition to the conventional BPC properties selected through a novel optimization strategy to predict the peptide status – amyloidogenic or non-amyloidogenic. The proposed approach achieves acceptable results, comparable to most online predictors. Besides, it compensates the lacuna in existing amyloid fibril prediction tools by maintaining equilibrium between sensitivity and specificity.

## Background

Amyloid-like fibrils may be formed from amylome, the universe of protiens. Today the association between protein fibrils and amyloid diseases, including Alzheimer’s and prion diseases has been established [1]. To find a solution for effective treatments of amyloid disorders, the fundamental problem of understanding the factors that stimulate conformational changes and aggregation in proteins need to be solved [2].

The inference that there is a predisposition for primary sequence-specific formation of amyloidal fibrils is made from the wet lab proven experimental remarks that not all proteins are amyloidogenic and that only precise continuous stretches of amyloid fibril forming peptides are more amyloidogenic than other regions. Furthermore, the observation that amyloids can be formed from short peptide fragments, seem to indicate that these segments, which are exposed to the environment, can cause the changeover of native proteins into amyloid state [2].

It is apparent that certain sequences have more amyloidogenity than others regardless of studies that seem to suggest that assembly into amyloid-like fibrils is an intrinsic property of peptides, irrespective of their sequence. Additionally, some short segment of peptides have same amyloid characteristics as full length proteins, and some fragments have been considered to be the regions causing aggregation, due to the fact that they can transform the amyloidogenic tendencies of polypeptides by favouring or obstructing the formation of fibrils. These data recommend that primary sequence can impact the formation of amyloid fibrils, and has stimulated the recent advancement of computational models to predict the amyloidogenic propensities of proteins [3].

The challenge of predicting amyloidogenic regions has resulted in a variety of multi-parametric methods that attempt to predict such motif sequences [4]. Each methodology has its own hypothesis and implements, ranging from simple to complex [5]. Overall, the success of different computational approaches in predicting aggregation-prone regions allow proposing that aggregation propensity in polypeptide chains is ultimately dictated by the sequence [6]. As research continues for the understanding of amyloid formation, the development of computational prediction techniques is an imperative supplement to experimental molecular approaches [5]. Several computational tools for predicting amyloid segments have emerged since 2004, such as [3,5-8] based on physicochemical grounds or structural denominators. However, methods by means of supervised machine learning models are only few such as Pafig [9].

In this article, we propose a supervised machine learning architecture that purely follows a sequence-based design strategy to determine the amyloidogenic short stretches in peptides. The systematically selected BPC properties of amino acids taken from Amino Acid index database in DBGet (Japan) and ProtScale in Swiss Expasy are utilized along with auto-correlation function and atomic composition within a peptide fragment to represent protein sequence features, and finally ANN is implemented to classify the fibril forming and non-fibril forming peptide segments. Prior to prediction, a feature optimization scheme based on evolutionary SVM is employed to search for the significant BPC features thereby reducing the dimensionality of the input space. The evolutionary strategy hybridizes a variant of Genetic Algorithm (GA) named Memetic Algorithm (MA) with SVM. The present study was initiated in an attempt to improve the overall performance in predicting the amyloid motifs in proteins by incorporating auto-correlation functions generated from selected amino acid indices and atomic composition of amino acids combined with corresponding BPC features obtained by a novel implementation of feature optimization.

## Results and discussion

Given the laborious nature of experimental validation of peptide segments most prone to form fibrils, it is imperative that computational approaches be developed that could produce reliable, affordable and testable *in silico* predictions [4]. By incorporating correlation of carefully selected amino acid indices through embedded SVM, among the residues within a window, we attempted to strengthen the sequence interaction effect in forming the feature vector thereby reducing the misclassifications. In fact, experimentally predicted amyloidogenic regions reported in different works do vary [10]. One possibility could be due to the fact that the sequences are examined under diverse conditions. Hence reliable identification of amyloid fibril stretches is challenging and difficult.

**Figure 1:**
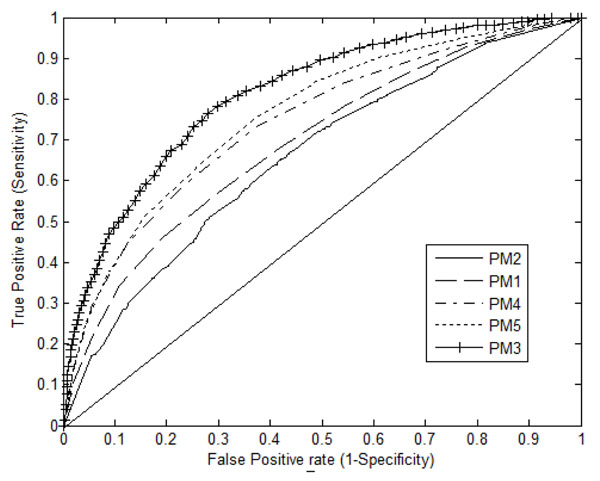
**ROC plot of the true positive rate versus the false positive rate of five prediction models trained with independent and integrated features.** The plot consists of five ROC curves on cross validation test (i) PM_1_ trained with 40 features (BPC properties) (ii) PM_2_ trained with 5 features (atomic compositions within a residue) (iii) PM_3_ trained with 65 features (40 BPC with their 25 autocorrelation function values) (iv) PM_4_ trained with 45 features (40 BPC with 5 atomic values) and (v) 70 features (40 BPC properties, 25 autocorrelation function values and 5 atomic compositions).

The cross validation and independent tests carried out on all five Prediction Models (PMs) showed that inclusion of feature like auto-correlation function significantly improved the sensitivity and specificity. Results of comparative studies on prediction models based on Sensitivity (S_n_) and Specificity (S_p_) are shown in Table 1. As seen, maximum performance is obtained by PM_3_ that integrates newly introduced correlation feature and the least by PM_2_ trained with constituent atoms alone. The performance of PM_3_ is further analysed using Receiver operating Characteristic (ROC) curves in figure 1. PM_3_ tested with BPC features and autocorrelation function values gives an Area Under Curve (AUC) of .854. The effect of other models with integrated and independent features have been investigated and found that the AUCs got significantly decreased as can be interpreted from figure 1. From these evaluations, we could establish that BPC features along with autocorrelation function are sufficient in determining the peptide status – amyloigogenic or non-amyloidogenic computationanlly.

**Table 1:** Performance of five prediction models in terms of Sensitivity (S_n_) and Specificity (S_p_)

	Cross validation test		Independent test	
Prediction models	S_n_	S_p_	S_n_	S_p_
PM_1_	74.4	72.8	70.7	69.3
PM_2_	61.6	59.3	55.4	52.3
PM_3_	82.3	80.2	77.8	80.1
PM_4_	75.5	74.6	72.6	71.9
PM_5_	78.2	75.4	76.4	74.7

### Comparison with existing approaches

The performance of the best PM is compared with two recently published prediction tools, FoldAmyloid [8] and AMYLPRED [5] which predict amyloidogenic regions from primary sequence, in terms of S_n_, S_p,_ Balanced Accuracy (BACC) [11] and Mathew’s Correlation Coeffecient (MCC). Prediction by FoldAmyloid is based on expected probability of hydrogen bond formation and expected packing density of residues and we chose the value of sliding window size and reliable frame size to be 6 to carry out the analysis. The tool AMYLPRED makes a consensus prediction of fibril forming regions in proteins by utilizing five diverse and individually published methods and the analysis was performed using the default parameters for each employed algorithm.

**Figure 2:**
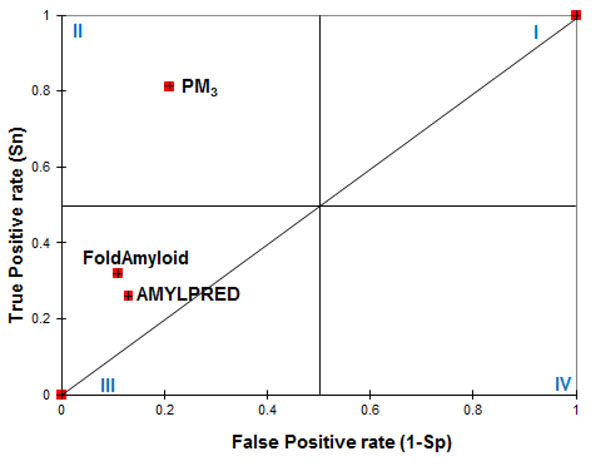
**Scatter plot of the true positive rates (sensitivity) versus the false positive rates (1-specificity) for various fibril prediction methods.** The plot consists of three points to illustrate that the proposed prediction model PM_3_ is superior in maintaining balance between true positive and false positive rates compared to other existing online tools.

In order to compare various methods, their performances are evaluated on the same dataset. As the online predictors included in our analysis were not assigned any prediction cutoff or threshold, ROC curves cannot be constructed completely for these tools. Therefore, their performances in terms of S_n_ and S_p_ are denoted by specific points on the ROC plot. Figure 2 shows the scatter plot for true positive rate (S_n_) versus false positive rate (1-S_p_) to compare the balance between S_n_ and S_p_ of the proposed method with other online tools. The plot area is split into four quadrants denoted I-IV as referred [12]. In fact, the four quadrants denote algorithm that achieves (i) higher S_n_ but lower S_p_ (ii) higher S_n_ and higher S_p_ (iii) lower S_n_ but higher S_p_ (iv) lower S_n_ and lower S_p_. The diagonal line (0, 0) – (1, 1) indicates a method that results in equal true positive rates and false positive rates. Hence, methods in quadrant II, far-off from the diagonal line are better performers.

Those methods that have its place in quadrant III have a tendency to predict all the examples as negative resulting in high specificity but very low sensitivity. FoldAmyloid and AMYLPRED appearing in quadrant III imply that although they have good specificity, (scores of .89 and .87 respectively), the sensitivity (scores of .32 and .26 respectively), is very poor. Out of these tools, AMYLPRED achieves the least BACC. MCC scores obtained by FoldAmyloid and AMYLPRED are .36 and .27 respectively.

Quadrant II in the plot is the best with both S_n_ and S_p_ being > 0.5. As evident from the plot, the best proposed prediction model is found in this quadrant. Our method shows the highest sensitivity with the optimum specificity than previously reported prediction tools. Remarkably, the prediction results of our method are moderate and it achieves S_n_ and S_p_ of 82.3% and 80.2% for cross validation test and 77.8% and 80.1% for independent test. The presented computational architecture (PM_3_) achieves the best BACC on an average of 80% and MCC score of .59. Although [8] and [5] show high specificity, the overall balanced accuracy is poor due to very low sensitivity. Nevertheless, the sensitivity of these tools decreased substantially for Amylhexset data or they suffered from highly biased prediction (very low sensitivity but very high specificity). Low sensitivity obtained by FoldAmyloid may be due to the fact that trans-membrane and signal regions are not considered in their study. Frousios et al., [5] reported that scores of .13 and .95 representing sensitivity and specificity respectively are obtained by AMYLPRED for a dataset consisting of 5006 data samples used in their work. Garbuzynskiy et al. [8] correctly predicted 80% and 72% of amyloidogenic peptides and non-amyloidogenic peptides respectively for a dataset of 407 peptides using FoldAmyloid webserver.

The statistical measures, S_n_ and S_p_, the equilibrium maintained between them in terms of balanced prediction accuracy and MCC across the test dataset indicate that our algorithm produces the most significant improvement. The influence of each separate feature may be lesser; however the progress on the performance of the algorithm is further apparent when these features were taken into account collectively. Instead of randomly choosing some physicochemical properties related to aggregation for training the model, we tried to get an optimized set of properties related to fibrillar aggregates using a supervised machine learning algorithm named evolutionary SVM through a novel implementation of the memetic algorithm. As reviewed [13] and discussed [14], structural information of peptides plays substantial role in determining amyloid fibrils. In fact, the selected BPC features include few amino acid indices related to protein structure study described in PROFEAT [15] which indicates that the presented models are trained with a feature subset that contains structural details of amino acid sequence as well. Though the results of our prediction model matched favourably with other methods, it needs to be enhanced further. Improvement in prediction results may be possible by identifying novel relevant features, features that maintain the sequence interaction effect and by incorporating more training data.

## Conclusion

The study of folding and unfolding events in proteins and subsequent aggregation into amyloid fibrillar deposits is becoming central to develop rational therapeutic strategies against devastations such as Alzheimer and Parkinson disease. A promising approach to spot such deposits is through computational prediction models. Even though, these models cannot replace the wet lab work, they might serve in identifying the regions of interest for further molecular investigations.

In our present study, due to the sheer amount of properties contained within the amino acids, we tried to provide a new and complementary set of physicochemical and biochemical properties through evolutionary SVM feature selection approach, besides their correlation and atomic composition within a residue in forming the feature vector to train the ANN model. To our knowledge, this is the first attempt to utilize auto-correlation function and atomic composition in computationally predicting the amyloidogenic or non-amyloidogenic peptide status. In addition, a variant and novel implementation of hybrid GA termed MA is implemented. Among the five prediction models built, PM_3_ - the one trained with 65 features gives the best results in terms of S_n,_ S_p_ and AUC of ROC curve which clearly provide indication that newly introduced feature, autocorrelation function which helps in maintaining the sequence order effect, besides the BPC properties selected through MA have high impact in determining the amyloid aggregates. As also evident from the MCC score and the scatter plot, the proposed *in silico* computing method achieves an agreeable result and preserves balance between the rates of true positives and false positives that is deficit in the existing online tools.

## Methods

### Sequence dataset gathering

The accuracy of fibril motif prediction can be enhanced with the use of soft computing approaches. However, the classifiers are required to be trained with appropriate datasets in the form of positive and negative data. We compiled experimentally proved proteins related to amyloidosis and proteins with no experimentally determined amyloidogenic regions published in literature [2,3,5-7,16,17], in order to construct the dataset (Additional file 1). The extracted sequences associated with protein aggregation include natively globular proteins, natively intrinsically unstructured proteins, amyloidogenic proteins and proteins related to depositional diseases. All protein sequences were downloaded in Fasta format from Uniprot-Swissprot database [18]. The wet lab analysis of different proteins reveals that these peptide sequences contain short stretches which form amyloid fibrils [8]. Thompson et al., [7] claim that hexpeptides are sufficient for forming amyloid-like fibrils. Therefore, a dataset of hexpeptides including positive and negative examples of fibril formation was prepared by sliding a window of six residues. We term this dataset Amylhexset. A dataset of 2512 hexpeptides of which 1232 that have been experimentally proved to form fibril forming segments and 1280 that have provided negative results in fibril forming assessment form Amylhexset.

### Feature encoding and mining

The overall capability of machine learning models to identify fibril aggregates is built on the encoded features of the dataset. ANN model needs each instance of data to be denoted in the form of real vectors. Therefore, the numerals of physiochemical or biochemical properties of amino acids in addition to their auto-correlation functions and atomic composition within a protein fragment are utilized to form the feature vector.

A collection of bio-physio-chemical characteristics of amino acids are proved to be supportive in studying protein macroscopic properties like aggregation, performing comparison among sequences or understanding conservation of functionally significant fragments in a peptide family (physio-chemical signatures). As these properties are proved to be useful in studying protein sequence profiles, folding and function, we have taken them into consideration for fibril motif identification. Moreover, computational approaches based on physio-chemical grounds have shown relatively good performances in predicting aggregation propensity [5,6,8,17]. The Amino Acid index (AAindex Version 9) [19] provides 544 characteristics for each of the 20 amino acids. Among the 544 indices, 13 were never considered due to partial data. According to Mathura et al., [20] properties with insufficient data and least relevant indices with respect to the study of protein structure, function and sequence are omitted in their database named APDbase [21]. Therefore, among all 531 features in AAindex database, only 246 are taken into account in APDbase. Of the 246 entries in APDbase, the last 29 entries (except MAXF760101 Normalized frequency of alpha-helix with description index 226 in APDbase) correspond to ProtScale in Swiss Expasy [22] which are not endowed with IDs or Accession Nos and the remaining 216 properties are from AAindex database. The authors have designated certain Accession Nos in a similar fashion as those of in AAindex version 9 for the very last 28 properties available in APDbase. Thus 531 in [19] + 28 in [22] indices were evaluated for potential use.

Experiments carried out by Goldscmidt et al., [1] suggest that if a fibrillizing sequence fragment is shuffled, the reordered segment loses its ability to form fibril aggregates. Keeping this in mind, a new feature namely auto-correlation function is introduced. Li et al., [15] suggest that auto-correlation feature describe the level of correlation between the amino acids in terms of their selected physico-chemical property within a residue. Moreau-Broto auto-correlation function of amino acid indices inspired from the work of [23] on pinpointing disordered regions in proteins is utilized. According to Han et al., [23] prediction accuracy may be improved by incorporating this function as it could test if the BPC property of an amino acid is dependent of that of its neighbours and has been used in the protein structural and functional classification studies. However, this was not effective in the prediction of membrane proteins [24]. The Moreau-Broto auto-correlation function *F_v_* of an amino acid index is calculated within a window, as:(1)

where *w* is the window size, *p_i_* and *p_i_*_+_*_v_* are the amino acid index values at positions *i* and *i* + *v* respectively [23]. Here, *w* = 6 and hence *F_v_*(*v* = 1,2,––,5) for the best 5 BPC properties selected through feature pre-optimization (discussed in the subsequent section) is calculated.

Atomic composition (AC) refers to Carbon, Hydrogen, Nitrogen, Oxygen and Sulphur atoms in an amino acid sequence. As the count of constituent atoms in each hexmer varies from one another, this feature is hypothesised to be a good choice as it helps in differentiating samples. Therefore, atomic values of samples in Amylhexset are included as five features in the encoding scheme. However, this feature does not contribute in maintaining the sequence order effect due to the fact that AC (hexmer) = AC (shuffled hexmer).

The values of each property were scaled so as to fall within a small specified range using min-max normalization technique [25]. Formulation of min-max normalization:(2)

Suppose that min*_P_* and max*_P_* are the minimum and maximum values of a feature, *P*, then min-max normalization maps a value, *v*, of *P* to *v*′ in the range [*new*_min*_P_*, *new*_max*_P_*] by computing equation (2). This transformation prevents features in greater numeric ranges from dominating those in smaller numeric ranges.

### Feature optimization

In order to attain a considerably improved performance in terms of classification ability, it is a prerequisite to generate relevant features so as to discriminate well among classes. One of the basic problems in computational biology is how effectively a lesser subset of significant features be selected [10]. Feature optimization involves two essential tasks: (i) feature pre-optimization [26]; and (ii) determining the best subset of features from pre-optimized set of features. The latter is achieved through evolutionary SVM, a method that is inspired from the work by Huang et al., [27] on protein subnuclear localization.

Filter based and embedded based models [28] were employed and evaluated for pre-optimization. In this regard, embedded model based on SVM classifier is found to be more effective in selecting an initial round of BPC properties and 186 properties are selected. The ultimate set of BPC properties are selected by evolutionary SVM utilizing a variant of hybrid GA termed MA. It is believed that one of the important factors affecting the GA results is due to the varying implementation of the GA method [10]. In this study, one such variation of hybrid GA resulting in MA is adopted.

**Figure 3:**
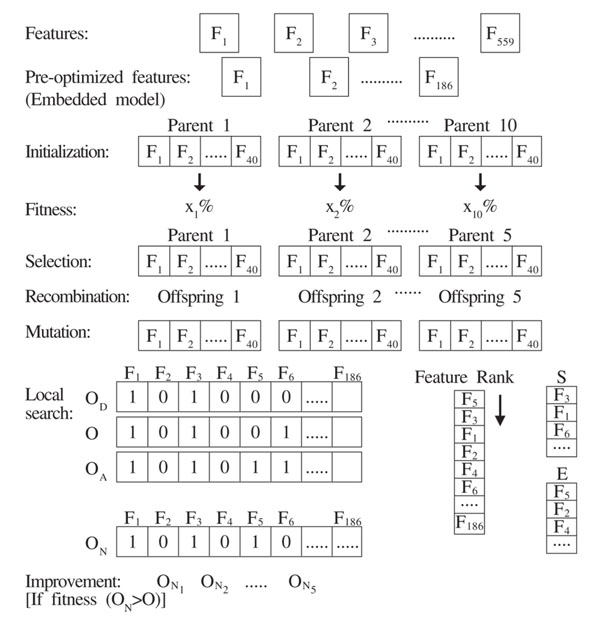
**Overview of feature optimization strategy.** This gives an overview of feature optimization strategy.

The proposed MA concisely presented in figure 3 showing the representation of feature vector of intermediate steps in the optimization procedure selects a subset out of 186 pre-selected features, and determines the corresponding SVM parameter values using 5-fold cross-validation as an estimator of generalization ability. For this purpose, LIBSVM [29] is trained with Amylhexset using Radial Basis Function (RBF) kernel.

**Figure 4:**
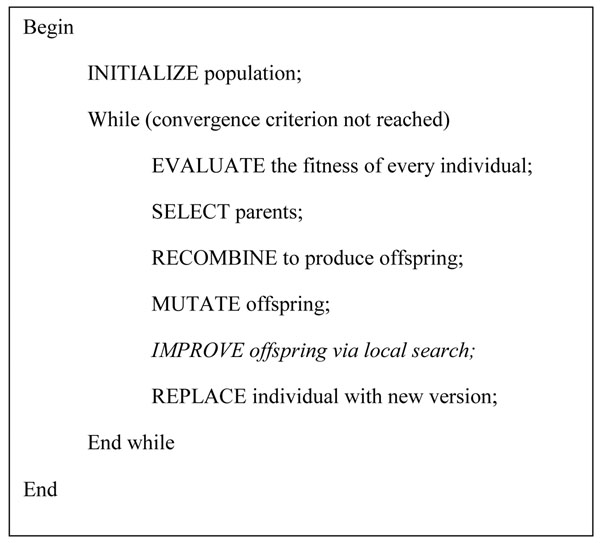
**Pseudo code for the memetic algorithm.** The pseudo code for the memetic algorithm is shown.

MA is an evolutionary strategy where elements of local optimization are incorporated in a conventional GA global search to increase the precision of the solution [30]. Eiben and Smith [31] suggest that local search acting on solutions created by mutation or recombination result in better solutions. Zhu et al., [32] propose a wrapper-filter dimensionality reduction methodology using a memetic outline. Predominantly, the approach adds or removes a feature from a feature subgroup based on their ranking. It has been shown in the literature that MA as an optimization technique utilizing filter ranking methods, result in the enhancement of classification performance. The pseudo code for MA is shown in figure 4. The major steps involved in MA are briefly discussed:

*Initialization*: Create an initial population with a set of randomly generated 10 parents with 40 properties each. This initial creation permits the GA to discover all possible range of solutions which support the most likely results to a great extent.

*Evaluation*: Compute fitness values of all individuals using SVM with 5-fold cross validation in the population and they are ranked according to their fitness.

*Selection*: With this approach, greater the fitness, higher the chance to move ahead to subsequent generation. To ensure that good individuals do survive to next generations, we choose the best half according to the fitness.

*Recombination*: A property pool array is defined to hold the shuffled properties of selected best parents. For every offspring, the properties in the pool were drawn one by one, saved if the property occurs the first time for the specific offspring else put back in to the pool. With this procedure it could be made sure, that a property which appeared more often in the fitter parents has a higher probability to be a part of the new generation.

*Mutation*: Mutation makes sure that the properties, which are not part of the first generation, have a chance to get into the algorithm later. A high mutation rate is desired for the first few generations, because it allows making big steps towards a better accuracy, but it should decrease with every generation to allow the algorithm to find the optimum with small changes. To set the number of mutations per offspring, we implemented an exponential function [10], depending on the number of actual generation.(3)

where N_M_ is the number of mutations per offspring during the actual generation n_G_, s is the size of offspring and m is the mutation value, a constant between 0 and 1 which defines the start value of the first generation depending on the size of an offspring. N_G_ stands for the total number of generations the algorithm is going to run for. In this work, the mutation value has been set to a value of 0.2 for every test.

*Improvement through local search*: Maintain an array of binary string of length equivalent to pre-optimized features for each offspring, so that each bit encodes a single feature. A bit of ‘1’ implies the corresponding feature is selected and a ‘0’ that is excluded.

Given an Offspring O, we define two sets S and E for Selected and Excluded properties represented in offspring respectively. Filter ranking method [32], F-statistic [10] has been used for ranking S and E with the most vital feature ranked the maximum score.

Two basic local search operators suggested by Zhu et al., [32] are defined namely (i) Add: Select a high ranked feature from E and add it to O resulting in O_A_ and (ii) Delete: Select a low ranked feature from S and delete it from O resulting in O_D_.

The new offspring O_N_ is formed by merging O_A_ and O_D_ such that the highly ranked features in E added to O_A_ are retained and the low ranked features in S excluded from O_D_ are removed. Since prior information on the optimum number of properties is known, the number of bit ‘1’ in each offspring is restricted to 40. Local search length defining the maximum count of Add and Delete procedures in each local search is set to a value 8.

For each mutated offspring O, O_N_ is created. Fitness functions for each pair of O and O_N_ are evaluated using LIBSVM. If the classification ability in terms of cross validation rate of O_N_ outperforms its corresponding O, then the mutated offspring O is replaced by O_N_. This improvement of offsprings through local search is continued for all mutated offsprings.

*Replacement*: A new population is formed by replacing the worst half discarded in selection process with the mutated improved offsprings.

*Convergence criterion*: The procedures of selection, recombination, mutation, local search and replacement continue till the convergence criterion is met which has been set to a maximum size of 100 generations.

The best combination of properties with cross validation rate of 83.34% was obtained in 89^th^ generation after which the accuracy remained constant. Therefore, 40 features acquired (Additional file 2) are utilized for feature vector representation.

### Building models on training data

In this contribution, five prediction models (PM_1_ – PM_5_) based on ANN are trained and built with the state-of-the-art implementation in Neural Network Pattern Recognition Tool of MATLAB R2008b that uses a two-layer feed-forward network with sigmoid output neurons. The PMs are trained with independent and integrated features such as (i) PM_1_ trained with 40 features (BPC properties) (ii) PM_2_ trained with 5 features (atomic compositions within a residue) (iii) PM_3_ trained with 65 features (40 BPC with their 25 autocorrelation function values) (iv) PM_4_ trained with 45 features (40 BPC with 5 atomic values) and (v) 70 features (40 BPC properties, 25 autocorrelation function values and 5 atomic compositions). Each PM has input nodes ranging from 5 to 70 depending on the dimension of the feature vector. The output layer of the model contains one unit with a target value ‘1’ if motif is positive or ‘0’ if motif is negative. The number of hidden layer units was selected as 23, by trial and error method. The network is trained with back propagation algorithm utilizing sigmoid transfer function as the activation function. The training data is partitioned into three subgroups. 60% of the total data were utilized to train the ANN. 20% each were used for validation and testing. In order to evaluate the performance of unobserved data that were not included in the training process, the model was further assessed by presenting another data subset comprising 1900 hexmer samples whose results are shown in Table 1.

## Competing interests

The authors declare that they have no competing interests.

## Authors' contributions

SSKN conceived the research, designed the code, and performed computational prediction, comparisons and statistical analysis. NVSR and HKS supervised the work and helped in manuscript preparation. All authors read and approved the final manuscript.

## Supplementary Material

Additional file 1 **Amylhexset** This file contains the Genbank / Swissprot Accession Nos. of positive and negative data samples collected from the literature, which have been used for training and testing. Click here for file

Additional file 2 **AAindex Ids or Accession Nos. of 40 BPC properties used.** This file shows the BPC properties selected by the memetic algorithm. Click here for file
