# Supplementary material for: Exploiting heterogeneous features to improve in silico prediction of peptide status – amyloidogenic or non-amyloidogenic
Source: BMC Bioinformatics. 2011 Nov 30;12(Suppl 13):S21. doi: 10.1186/1471-2105-12-S13-S21 (PMC3278838; doi:10.1186/1471-2105-12-S13-S21)
Supplement: Additional file 1 — Amylhexset This file contains the Genbank / Swissprot Accession Nos. of positive and negative data samples collected from the literature, which have been used for training and testing. [file 1471-2105-12-S13-S21-S1.pdf]

Additional file 1 – Amylhexset

This file contains the Genbank / Swissprot Accession Nos. of positive and negative protein sequences collected from literature. Hexmers were then obtained using a six-residue sliding window as per the experimentally proved regions. Among the positive and negative data, P37840, P04156, P68082, P00698, P04279 and P01034, P01857, P01625, P12969 respectively have been used for independent testing.

Positive data

1. P02766
2. P37840
3. P04156
4. P02647
5. P19707
6. P10997
7. P01236
8. P01258
9. P02663
10. P02735
11. P02788
12. P04279
13. P06396
14. P10636
15. P22398
16. P61626
17. Q08431
18. P19707
19. P05067
20. P68082
21. P00698
22. Q9Y287
23. P05453
24. P02655
25. P11686
26. P02671
27. P15502
28. P03036
29. P02185

30. P02247
31. P00287
32. P00974
33. P22303
34. P02846
35. P02848

Negative data

1. P01625
2. P00441
3. P01857
4. P01034
5. P12969
6. P01308
