# Supplementary material for: Exploiting heterogeneous features to improve in silico prediction of peptide status – amyloidogenic or non-amyloidogenic
Source: BMC Bioinformatics. 2011 Nov 30;12(Suppl 13):S21. doi: 10.1186/1471-2105-12-S13-S21 (PMC3278838; doi:10.1186/1471-2105-12-S13-S21)
Supplement: Additional file 2 — AAindex Ids or Accession Nos. of 40 BPC properties used. This file shows the BPC properties selected by the memetic algorithm. [file 1471-2105-12-S13-S21-S2.pdf]

Additional file 2 – AAindex Ids or Accession Nos. of 40 BPC properties used.
This file shows the BPC properties selected by Memetic Algorithm.

AGGR000246 (Aggregation Potential)	KUHL950101 (Hydrophilicity scale)
BHAR880101 (Average flexibility indices)	LEVM780102 (Normalized frequency of beta-sheet, with weights)
BULH740101 (Transfer free energy to surface)	MEIH800101 (Average reduced distance for C-alpha)
BURI000230 (Molar fraction (%) of 2001 buried residues)	MEIH800102 (Average reduced distance for side chain)
CHAM810101 (Steric parameter)	MIYS850101 (Effective partition energy)
CHOC760102 (Residue accessible surface area in folded protein)	MONM990201 (Averaged turn propensities in a transmembrane helix)
CHOP780203 (Normalized frequency of beta-turn)	NADH010101 (Hydropathy scale based on self-information values in the two-state model (5% accessibility))
CHOP780211 (Normalized frequency of C-terminal non beta region)	NADH010103 (Hydropathy scale based on self-information values in the two-state model (16% accessibility))
CIDH920105 (Normalized average hydrophobicity scales)	NAGK730101 (Normalized frequency of alpha-helix)
DESM900101 (Membrane preference for cytochrome b: MPH89)	NAKH900110 (Normalized composition of membrane proteins)
ENGD860101 (Hydrophobicity index)	NAKH920105 (AA composition of MEM of single-spanning proteins)
FAUJ830101 (Hydrophobic parameter pi)	NAKH920108 (AA composition of MEM of multi-spanning proteins)
FUKS010108 (Interior composition of amino acids in nuclear proteins)	PONP800101 (Surrounding hydrophobicity in folded form)

GUYH850101 (Partition energy)	PRAM900101 (Hydrophobicity)
GUYH850104 (Apparent partition energies calculated from Janin index)	PRAM900103 (Relative frequency in beta-sheet)
NORM000219 (Normalized consensus hydrophobicity scale)	PUNT030102 (Knowledge-based membrane-propensity scale from 3D_Helix in MPtopo databases)
JACR890101 (Weights from the IFH scale)	ROSG850102 (Mean fractional area loss)
JANJ780103 (Percentage of exposed residues)	ROSM880102 (Side chain hydropathy, corrected for solvation)
JANJ790101 (Ratio of buried and accessible molar fractions)	VHEG790101 (Transfer free energy to lipophilic phase)
JANJ790102 (Transfer free energy)	VINM940104 (Normalized flexibility parameters (B-values) for each residue surrounded by two rigid neighbours)
